# Feature Extraction of Surface Electromyography Using Wavelet Weighted Permutation Entropy for Hand Movement Recognition

**DOI:** 10.1155/2020/8824194

**Published:** 2020-11-24

**Authors:** Xiaoyun Liu, Xugang Xi, Xian Hua, Hujiao Wang, Wei Zhang

**Affiliations:** ^1^School of Artificial Intelligence, Hangzhou Dianzi University, Hangzhou 310018, China; ^2^Key Laboratory of Brain Machine Collaborative Intelligence of Zhejiang Province, Hangzhou 310018, China; ^3^Jinhua People's Hospital, Jinhua 321000, China; ^4^Hangzhou Vocational & Technology College, Hangzhou 310018, China

## Abstract

The feature extraction of surface electromyography (sEMG) signals has been an important aspect of myoelectric prosthesis control. To improve the practicability of myoelectric prosthetic hands, we proposed a feature extraction method for sEMG signals that uses wavelet weighted permutation entropy (WWPE). First, wavelet transform was used to decompose and preprocess sEMG signals collected from the relevant muscles of the upper limbs to obtain the wavelet sub-bands in each frequency segment. Then, the weighted permutation entropies (WPEs) of the wavelet sub-bands were extracted to construct WWPE feature set. Lastly, the WWPE feature set was used as input to a support vector machine (SVM) classifier and a backpropagation neural network (BPNN) classifier to recognize seven hand movements. Experimental results show that the proposed method exhibits remarkable recognition accuracy that is superior to those of single sub-band feature set and commonly used time-domain feature set. The maximum recognition accuracy rate is 100% for hand movements, and the average recognition accuracy rates of SVM and BPNN are 100% and 98%, respectively.

## 1. Introduction

Surface electromyography (sEMG) is an electrophysiological signal produced by a motor unit during the muscle activities of the human body. Given its advantages of being a noninvasive and straightforward operation, sEMG is widely used as the ideal biological signal control source for bionic rehabilitation equipment and new human-computer interaction equipment [1–4]. Due to accidents, diseases, and other reasons, many patients lose their hands or part of their arms every year. The lack of upper limbs or dysfunction has brought many inconveniences and troubles to the daily life of the disabled. The research of collecting sEMG signals from specific muscle groups to recognize hand movements has gradually emerged, and many studies have been made in the field of intelligent prostheses [5]. The hand-action pattern recognition technology based on sEMG is deeply studied to obtain high-efficiency and accurate hand-action recognition ability, and the recognition results are converted into multi-degree-of-freedom control instruction to drive the output [6], which has very important research significance and application value for the research of intelligent artificial hand.

As a typical application of sEMG, a myoelectric prosthetic hand [4] is controlled by sEMG signals produced by the amputee's residual muscles. With the development of science and technology, more and more new technologies and ideas have been integrated into the application of artificial limbs [7–9]. Due to the complexity of sEMG and the diversity of hand movements, there are still many problems and challenges to achieve efficient and accurate action analysis [10], such as the selection and extraction of sEMG features and the limited type and quantity of recognition actions. Many organizations and research institutions have exerted considerable effort in the feature extraction of sEMG signals to improve recognition accuracy because such accuracy is highly dependent on extracted features [11–14]. Feature extraction, which is related to the quality of pattern recognition, is the key to analysis and processing of sEMG. Previous studies used popular feature extraction methods in the time or frequency domain, such as root-mean-square (RMS) [15] and median frequency [16], to extract sEMG features. In the face of increasingly diverse feature extraction methods, the current EMG feature extraction methods only provide limited neural/motor information. Few features can fully reflect the detailed characteristics of sEMG signals [17]. The major problem is extracting useful information from signals [18, 19].

Wavelet transform (WT) [20] is a time-frequency analysis technology that exhibits multiresolution characteristics. It has the ability to characterize the local features of signals in the time and frequency domains. Duan et al. [21] used the WT method to extract time and frequency information for analyzing hand movements and achieved good results. Most studies provide limited information regarding wavelet processing and signal reconstruction but disregard the recognition research of wavelet sub-bands. Subasi et al. [22, 23] used discrete WT (DWT) to decompose sEMG signals into orthonormal time series with different frequency bands for feature extraction. The results showed that changes in sEMG signals can be effectively represented by features extracted from each sub-band of DWT. In [19, 23, 24], wavelets were applied to the preprocessing and decomposition of sub-bands. These studies similarly showed that calculating the sub-band parameter is useful in motion detection and other same application. Among the various characteristic parameters, entropy is an index used to measure the complexity of a system. The traditional entropy only considers the probability distribution of the signal value and does not consider the order structure of the signal value. Although there is a different definition for entropy and the way to calculate it, all kinds of entropies show system randomness and regularity [25]. As a nonlinear dynamic parameter based on complexity measurement, permutation entropy (PE) has been gradually applied to the analysis of complex bioelectrical signals. It can describe the local structure features of time series and enlarge the subtle changes in the signals with low complexity and antinoise ability [19]. Considering the complexity of EMG signals in various hand movements, PE can be used to reflect its intrinsic complex characteristics. However, in addition to the sequence structure, another disadvantage of PE is the possible loss of considerable information existing in the amplitude of a time series [26]. Fadlallah [27] presented weighted permutation entropy (WPE), which can extract the sequence structure of a time series and retain its amplitude information. From structural feature representation, WPE can extract local microstructure features, and wavelet transform can extract global macroscopic structural features. Thus, the combination of wavelet sub-bands analysis and WPE can comprehensively describe the features and can be an effective method for recognizing sEMG signals. In this study, a wavelet weighted permutation entropy (WWPE) method was proposed for hand action recognition.

## 2. Materials and Methods

### 2.1. Data Acquisition

**Figure 1:**
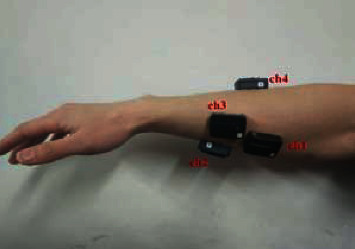
Target placement of sEMG sensors.

**Figure 2:**
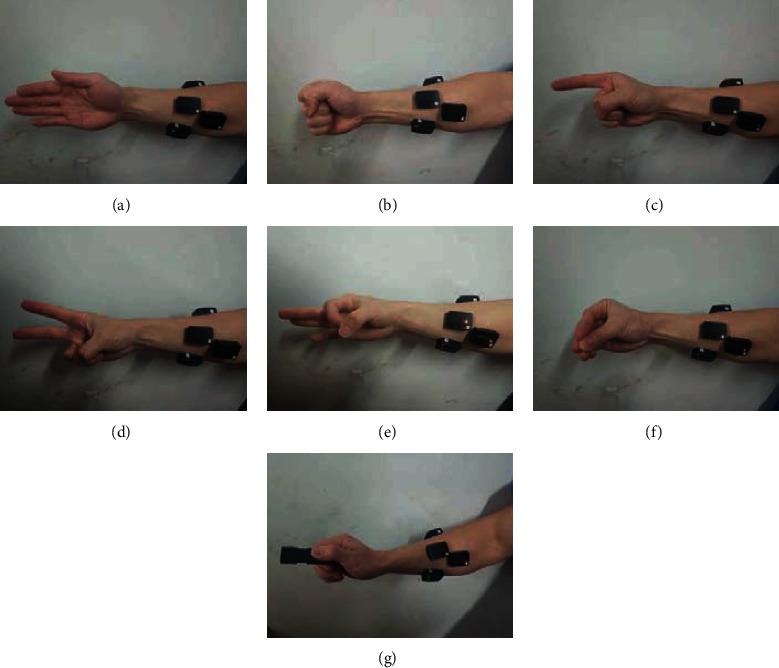
Hand movements: (a) open, (b) close, (c) point, (d) yeah, (e) ok, (f) tripod, and (g) grip.

Four healthy subjects (two males and two females; age: 24–26 years; height: 160–180 cm; weight: 48–70 kg) participated in the experiments by performing the designated hand movements. All the subjects read and signed an informed consent form approved by an institutional review board. Prior to data acquisition, the subjects' skin was wiped with alcohol, and then the subjects were asked to seat with their arm straightened and fixed at one position to avoid the effect of different limb positions on sEMG signals. Sensors were placed on each subject's flexor carpi radialis (ch1), flexor digitorum superficialis (ch2), flexor pollicis longus (ch3), and extensor digitorum (ch4) to record four channels of sEMG signals (Figure 1). Then, the following seven daily hand movements were performed: open, close, point, yeah, ok, tripod, and grip (Figure 2). The seven daily hand movements are more commonly used in daily life [28]. The four sensors used were Trigno™ Wireless EMG (Delsys Inc., Natick, MA, USA). These sensors provide motion artifact suppression (patent) that can be freely moved, and they directly transmit data wirelessly. The parameters of these sensors are as follows: resolution, 16 bit; bandwidth, 20–450 Hz; baseline noise, <1.25 *μ*V (RMS); typical operating range, 40 m; and communication protocol, Bluetooth®. In the experiments, the participants repeated each action 30 times. Each movement lasted for 3 s, and the total number of each movement should be at least 120. sEMG signals were recorded at a sampling frequency of 1000 Hz using EMGworks® 4.0 (Delsys Inc., Natick, MA, USA).

### 2.2. Algorithm Flowchart

**Figure 3:**
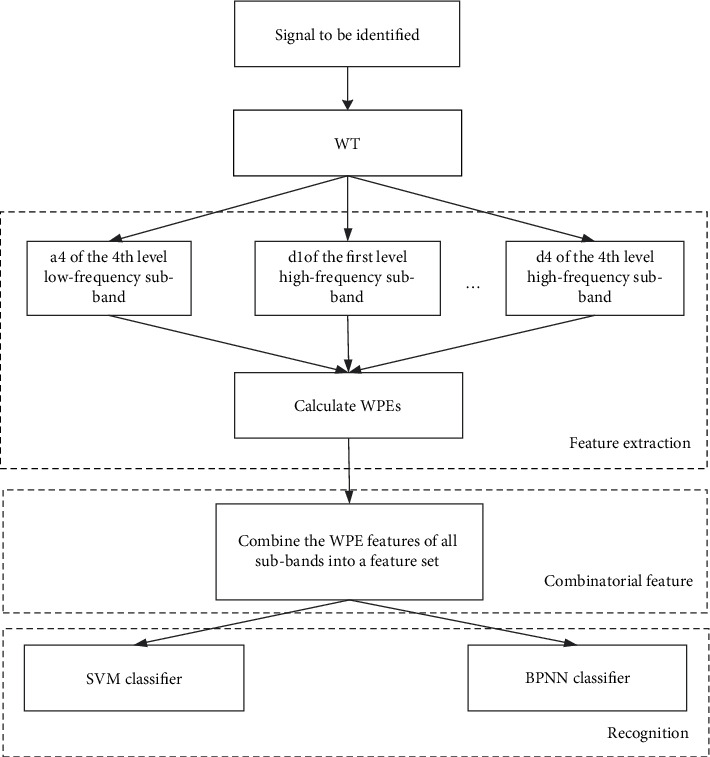
Algorithm flowchart.

The method proposed in our study for recognizing the seven basic hand movements using sEMG includes the following steps: wavelet decomposition, feature extraction, combinatorial features, and classification. The steps are illustrated in detail in Figure 3. After collecting sEMG signals, the feature vectors of the sEMG signals should be extracted. Considering the advantages of time-domain and frequency-domain analyses, WT was used to preprocess and analyze sEMG signals. The WPEs of wavelet sub-bands were extracted on the basis of the wavelet decomposition. WWPE is WPEs of the wavelet sub-band signal under wavelet decomposition. Lastly, the feature set was used as input to the SVM and BPNN classifiers to realize the pattern recognition of sEMG signals.

### 2.3. Wavelet Decomposition

**Figure 4:**
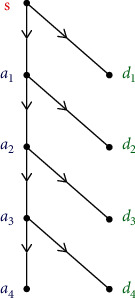
Decomposition of sEMG sequence via four-level WT and extraction of five sub-bands. “a4” is the approximation at the fourth level. “d4” is the details at the fourth level. “d3” is the details at the third level. “d2” is the details at the second level. “d1” is the details at the first level.

WT is a powerful time-frequency approach for biosignals. In our study, sEMG signal is decomposed into wavelet sub-bands in each frequency segment using WT. The efficiency of WT decomposition is dependent on the appropriate selection of the mother wavelet function [21]. The recognition accuracy of the signal depends on the similarity between the mother wavelet function and the wavelet coefficients [29]. Before selection of wavelet sub-band, the maximum energy-to-Shannon entropy ratio criterion is used for wavelet base selection. More detailed information can be found in [19]. Daubechies (DbN) are orthogonal and asymmetrical compact support functions [30]. Symlet (SymN) is an improvement of DbN; it can reduce phase distortion in signal analysis or reconstruction to a certain extent [31]. Sym8 possesses nearly similar attributes that match well with those of biosignals. sEMG signals were recorded at a sampling frequency of 1000 Hz using EMGworks® 4.0 (Delsys Inc., Natick, MA, USA). A sampling theorem is used to obtain the maximum frequency of 500 Hz. Since sEMG signal energy is concentrated in 30–200 Hz, according to the frequency distribution, in this paper, four-layer wavelet decomposition is carried out. Thus, sEMG signals were analyzed and processed via four-layer wavelet decomposition with “sym8” as the mother wavelet. As shown in Figure 4, we extracted five wavelet sub-bands (i.e., a4, d1, d2, d3, and d4) to achieve better results in feature extraction with WPE algorithm. Here, “a” represents low frequency, “d” represents high frequency, and the number represents the number of decomposition layers. Now, we can calculate WPEs for each sub-band in the next level.

### 2.4. Weighted Permutation Entropy (WPE)

PE is primarily used to measure the complexity of chaotic signals. The calculation, which is based on the comparison of adjacent data, is simple. Its antinoise ability is strong, and it exhibits good robustness.

Given a time series {*x*(*i*),  *i* = 1,2,   …,  *N* − (*m* − 1)} with length *N*, the *m*-dimensional embedding vector at time *i* is defined as

(1) $$\begin{array}{c} X_{i}=\;\left\{x\left(i\right),\;x\left(i+\tau \right),\ldots ,\;x\left(i+\left(m-1\right)\tau \right)\right\}, \end{array}$$

where *τ* is the delay time and  *m* is the embedding dimension. The time delay  *ττ* is related to the sampling rate of the signal. As [32] suggests, the time delay *τ* is set to 1 and the embedding dimension  *m* is set to 4 in this paper.

The *i-*th reconstructed components are rebuilt in the ascending order as follows:

(2) $$\begin{array}{c} x\left(i+\left(j_{1}-1\right)\tau \right)\leq x\left(i+\left(j_{2}-1\right)\tau \right)\leq \ldots \leq x\left(i+\left(j_{m}-1\right)\tau \right). \end{array}$$

If *X*_*i*_ has the same element, it can be sorted in accordance with the size of *j*. In other words, when  *x*(*i*+(*j*_*m*1_ − 1)*τ*)=*x*(*i*+(*j*_*m*2_ − 1)*τ*) and *j*_*m*1_ < *j*_*m*2_, the sorting method is

(3) $$\begin{array}{c} x\left(i+\left(j_{m1}-1\right)\tau \right)\leq x\left(i+\left(j_{m2}-1\right)\tau \right).\; \end{array}$$

Hence, any vector *X*_*i*_ can obtain a sequence of symbols as follows:

(4) $$\begin{array}{c} \pi_{j}=\left(j_{1},\;j_{2},\ldots ,\;j_{m}\right). \end{array}$$

Different sequences of symbols ( *j*_1_,  *j*_2_,…,  *j*_*m*_) have a total of m! sequences of symbol.

For a permutation with number *π*_*j*_, let *f*(*π*_*j*_) denote the frequency of the *j-th* permutation in the time series. Then, the probability of occurrence of each symbol sequence is calculated as follows:

(5) $$\begin{array}{c} p_{j}\left(\pi_{j}\right)=\frac{f\left(\pi_{j}\right)}{\sum_{j=1}^{m!}f\left(\pi_{j}\right)}. \end{array}$$

The PE of different sequences of symbols is defined as follows:

(6) $$\begin{array}{c} \;H_{P}\left(m\right)=-\sum_{j=1}^{m!}p_{j}\left(\pi_{j}\right)\ln\;p_{j}\left(\pi_{j}\right). \end{array}$$

PE provides a measure of the complexity of a nonlinear time series represented by the sequential patterns. However, in accordance with its definition, PE disregards the amplitude differences between the same sequential patterns and loses the information regarding signal amplitude.

On the basis of the difference in amplitude or variance of a certain mode, WPE assigns a weight value to each extracted vector when calculating the relative frequency associated with each symbol. The weight value  *ω*_*j*_ is calculated by the variance of each subsequence *X*_*i*_. *ω*_*j*_ is expressed as

(7) $$\begin{array}{c} \omega_{j}=\frac{1}{m}\sum_{k=1}^{m}{\left(x\left(i+\left(k-1\right)\tau \right)-\bar{X_{i}}\right)}^{2}, \end{array}$$

where $\bar{X_{i}}$ is the arithmetic mean as follows:

(8) $$\begin{array}{c} \bar{X_{i}}=\frac{1}{m}\sum_{k=1}^{m}x\left(i+\left(k-1\right)\tau \right). \end{array}$$

For a permutation with number *π*_*j*_, the frequency of the *j*-th permutation in the time series can be defined as

(9) $$\begin{array}{c} f_{\omega }\left(\pi_{j}\right)=\;\sum_{s}^{S}f\left(\pi_{j}\right)\omega_{j}, \end{array}$$

where *s*=1,2,…, *S* and *S* is the number of the possible time series in the same ordinal pattern. The weighted relative probability of occurrence for each symbol is

(10) $$\begin{array}{c} p_{\omega }\left(\pi_{j}\right)=\frac{f_{\omega }\left(\pi_{j}\right)}{\sum_{j=1}^{m!}f_{\omega }\left(\pi_{j}\right)}. \end{array}$$

WPE is defined as follows:

(11) $$\begin{array}{c} H_{\omega }\left(m\right)=-\sum_{j=1}^{m!}p_{\omega }\left(\pi_{j}\right)\ln\;p_{\omega }\left(\pi_{j}\right).\; \end{array}$$

In this study, we recorded 4-channel EMG signals using Trigno™ Wireless EMG (Delsys Inc, Natick, MA, USA) and proposed the feature extraction of sEMG signals using a wavelet weighted permutation entropy (WWPE) method for recognizing seven hand movements based on four channels. We used the sEMG and its sub-bands extracted from the wavelet decomposition as inputs for WPE algorithm and calculated WPE values for each sub-band. The WPE of a single wavelet sub-band is a 4-dimensional feature set. WWPE is WPEs of the wavelet sub-band signal under wavelet decomposition (i.e., a4, d1, d2, d3, and d4), which is a 20-dimensional feature set. In other words, the WPEs of all sub-bands were combined into a feature set as WWPE feature set. Then, the WWPE feature set was used as the input to the SVM and BPNN classifiers to realize the pattern recognition of sEMG signals.

## 3. Results

### 3.1. Wavelet Decomposition

**Figure 5:**
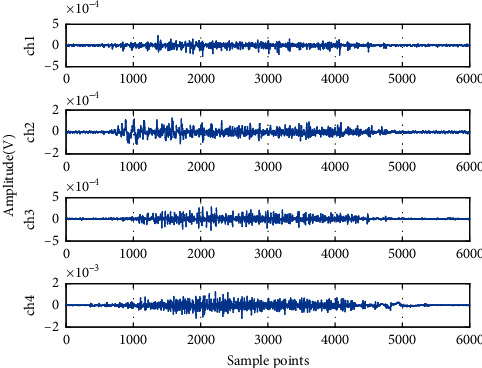
Raw sEMG signals.

**Figure 6:**
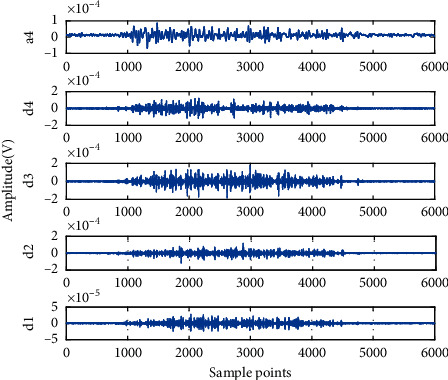
Five-layer wavelet decomposition signal of ch3.

**Figure 7:**
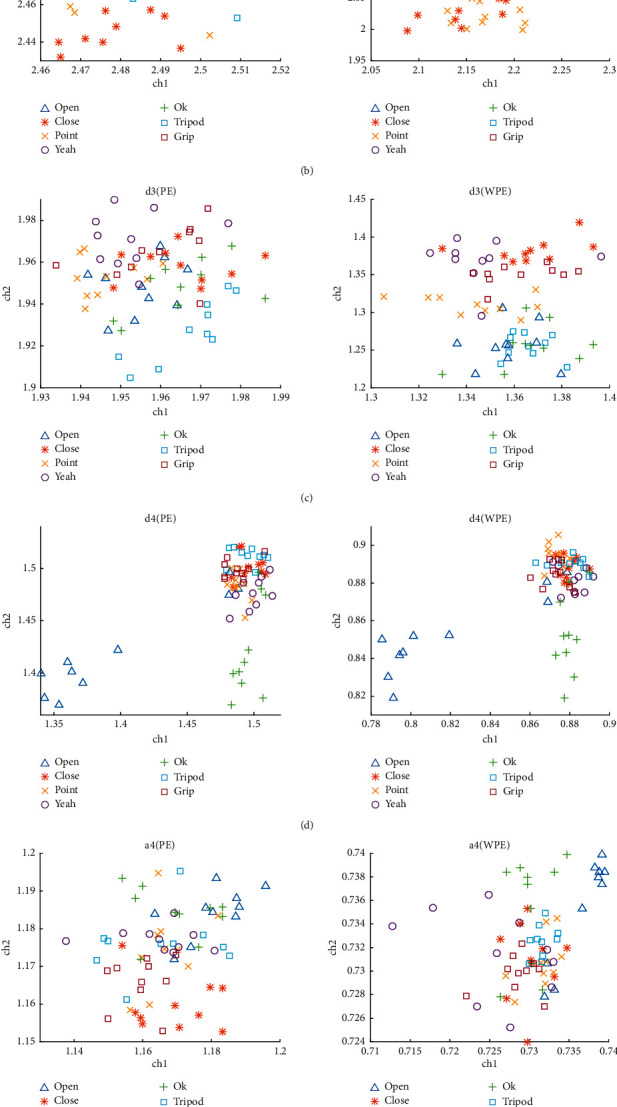
Scatterplots of the entropy values of the random two-channel sEMG signals for the seven hand movements: (a) d1, (b) d2, (c) d3, (d) d4, and (e) a4 wavelet sub-bands and (f) undecomposed sEMG. The horizontal coordinate represents entropy values of sEMG from ch1, and the longitudinal coordinate refers to those from ch2.

The four-channel raw sEMG signals of one set of hand movements acquired from one subject are shown in Figure 5. The horizontal coordinate represents the number of sample points, and the longitudinal coordinate represents the amplitude of sEMG signals. In this figure, ch1, ch2, ch3, and ch4 represent flexor carpi radialis, flexor digitorum superficialis, flexor pollicis longus, and extensor digitorum shown in Figure 1, respectively. WT was used to decompose and preprocess sEMG signals to obtain the wavelet sub-bands in each frequency segment via four-layer wavelet decomposition with “sym8” as the mother wavelet. The five wavelet sub-band signals (i.e., a4, d1, d2, d3, and d4) of ch3 were extracted as shown in Figure 6. The horizontal coordinate represents the number of sampling points, and the longitudinal coordinate represents the amplitude of signals. As clearly indicated in Figure 7, the different wavelet sub-bands have disparate profiles because they are carrying varying degrees of effective information. The sEMG signals of the other channels and subjects exhibit similar traits. Therefore, calculating wavelet WPE as a feature for discriminating different movements is possible.

### 3.2. Feature Analysis

For a qualitative observation of the feature distributions of the seven hand movements, the WPE and PE distributions in ch1 and ch2 randomly selected from one of the subjects are presented in Figure 7. In this figure, the horizontal coordinate represents entropy values of sEMG from ch1, and the longitudinal coordinate refers to those from ch2. This figure shows that a clear distinction between the points of the seven movements is barely visible; nevertheless, observing that WPE is slightly better than PE in clustering is still possible. In addition, the distributions of points in Figures 7(b), 7(c), and 7(f) are considerably clearer, and the boundaries and points of different movements are more evident. The WPEs of d2 and d3 achieve significantly better clustering performance than those of the other sub-bands. Compared with the PE and WPE of sEMG, the clustering performance of the d2 and d3 sub-bands is nearly as good as that of sEMG. The results show that the features extracted by the wavelet sub-band can be used to distinguish the action. Thus calculating the sub-band parameters is useful in motion detection.

### 3.3. Recognition Accuracy

Three-layer BPNN and SVM were used as classifiers in the experiments. We divided all the samples into two sets. Some samples are randomly selected from all the training data as the training set and the rest as the test set. To illustrate the identification performance of PE and WPE on different sub-bands (i.e., a4, d1, d2, d3, and d4), all PEs and WPEs were used as input to be recognized by SVM and BPNN.  Table 1 provides the average recognition accuracies of different sub-bands and sEMG under seven movements. WPE achieves better recognition accuracy than PE in SVM and BPNN. The WPE of d3 exhibits considerably better identification performance than those of the other sub-bands. The average recognition accuracy of d3 reaches 87%, which is approximately 10% higher than those of the other sub-bands. In addition, no significant difference in accuracy is observed between d3 and sEMG in the two classifiers.  Table 2 provides the detailed identification results of WPE and PE from the d3 sub-band and undecomposed sEMG under seven movements. The WPE of the d3 wavelet sub-band can distinguish among each hand movement with high precision that can reach up to 100%. These results validate the rationality of extracting features from wavelet sub-bands for recognition.

In this study, a wavelet weighted permutation entropy (WWPE) method was used for recognition.  Table 3 provides the average recognition accuracies of features. However, the WPE of d3 exhibits considerably better identification performance than those of the other sub-bands in Table 1. The average recognition effect of proposed method is considerably better than the single d3 wavelet sub-band feature. The average recognition accuracy of WWPE feature set is better. It can be seen from Table 3 that the WWPE feature set could provide higher classification accuracy that reaches 100% and approximately 98% in SVM and BPNN, respectively. To further illustrate the superiority of the WWPE feature set presented in Table 3, the identification performance of the method above is compared with the signals in Table 1. Compared with that in Table 1, the method we proposed in Table 3 can result in an increase of approximately 15% in average identification accuracy. The detailed recognition results of hand movements are provided in Tables 2 and 4. The same conclusion can be drawn that WWPE feature set is considerably better in terms of recognition performance. Although the d3 wavelet sub-band is comparable with sEMG in terms of performance, the WWPE feature set is the best. The WWPE feature set obtains the highest recognition accuracy because WWPE feature set can explore more information than a single wavelet sub-band feature set. The experiment results validate that the proposed method based on a WWPE feature set can achieve high identification accuracy. The feature extraction of sEMG using WWPE for hand movement recognition exhibits a remarkable advantage.

The traditional time-domain feature set, consisting of root-mean-square (RMS), mean absolute value (MAV), waveform length (WL), zero crossings (ZC), and slope sign changes (SSC), was compared with our proposed features. The recognition accuracies of traditional feature set are shown in Table 5. We can see that the proposed method has considerable success in the activity classification. Compared with Table 2, wavelet sub-band recognition accuracy of some action is better than the traditional feature set. For OK action, the accuracy of d3 wavelet sub-band is greater than 85%. A similar situation exists for other movements. These results validate the rationality of extracting features from wavelet sub-bands for recognition. The proposed method based on WWPE achieves the highest identification accuracy.

## 4. Discussion

Four healthy subjects participated in the experiments for the seven hand movements. Wavelet analysis was combined with entropy features. WT was used to decompose and preprocess sEMG signals to obtain wavelet sub-bands. Then, the WPEs of the wavelet sub-bands were extracted. WWPE is WPEs of the wavelet sub-band signal under wavelet decomposition. To further illustrate the superiority of the WWPE feature, its recognition performance was compared with those of WPE and PE of sEMG, WPE, and PE of single wavelet sub-band and the wavelet PE feature set. In our experiment on pattern recognition using sEMG signals, only overall recognition accuracy was considered.

The detailed results are presented in Tables 2, 4, and 5. The result shows that the proposed method achieves good performance, and recognition accuracy can reach up to 100% for each hand movement. For the wavelet sub-bands in Table 1, the average identification accuracy of the d3 sub-band can be nearly as good as that of undecomposed sEMG. A d3 wavelet sub-band can distinguish among the hand movements with high precision (87%). The maximum accuracy for movement can reach up to 100% in Table 2. Although the d3 wavelet sub-band exhibits good recognition accuracy, the WWPE feature set achieves the higher recognition accuracy and can reach 100% in SVM and 98% in BPNN. Most current practices based on hand motion recognition often adopt the idea of combining more time-domain features to identify limb movements for improved classification performance [16]. Five commonly used time-domain features (RMS, MAV, WL, ZC, and SSC) extracted and combined from sEMG were compared with our proposed feature. The experiment results validate that the proposed method based on WWPE can achieve high identification accuracy than time-domain feature set. In addition, the wavelet PE and the WWPE constructed by the same idea have similar recognition results for the seven movements. However, it is undeniable that this method can achieve high recognition accuracy. They are more effective than single wavelet sub-bands and traditional time-domain feature set.

Most studies provide limited information on wavelet processing and signal reconstruction but ignore wavelet sub-band recognition. Wavelet transform brings macrostructure information into the feature. The macro- and microstructure information and amplitude information are all explored by WWPE. Combined with macro- and microstructure information, WWPE can get better performance. Therefore, the combination of wavelet sub-band analysis and WPE can comprehensively describe the characteristics and can effectively describe the non-stationary and nonlinear characteristics of sEMG signals. It proves the rationality of extracting features from wavelet sub-bands for recognition. The combination of wavelet sub-band analysis and entropy can be an effective method to identify sEMG signals.

SVM and BPNN have good learning and generalization abilities. Regardless of which features, SVM and BPNN present varying recognition results. However, the difference between them is minimal. Consequently, the method based on WWPE for hand movements demonstrates high potential in the future.

The limitations of this work cannot be denied, Similar to most other methods, we do not have a database that is applicable to all individuals. We limited our experimental subjects to healthy people instead of amputees. Future work can focus on creating a large database of hand movements with multiple action categories and consider recruiting amputees to further validate the performance of their proposed method because amputees are the target users of the prosthetic devices rather than considering only healthy subjects. In addition, other methods are also likely to achieve higher classification accuracy by using more computing resources. This issue should be the focus of future research.

## 5. Conclusions

Feature extraction, which is related to the quality of pattern recognition, is the key to analysis and processing of sEMG. This study proposes the feature extraction of sEMG signals using WWPE for recognizing seven hand movements based on four channels. We applied WT to preprocess and analyze sEMG signals. Then, the WPEs of sEMG and its wavelet sub-bands were extracted to construct WWPE feature set. A support vector machine (SVM) and a back-propagation neural network (BPNN) were used to classify hand movements. The validity of sEMG and its wavelet sub-bands in recognizing daily hand movements was calculated. The experimental results show that the WWPE feature set extracts more comprehensive feature information and achieves the highest identification accuracy. Furthermore, the WWPE feature set is superior to the single sub-band feature and commonly used time-domain feature set. It is proved that it is reasonable to extract features from wavelet sub-band for recognition. A combination of wavelet sub-band analysis and entropy can be an effective method to identify sEMG signals and be effectively applied to pattern recognition of hand movements.

## Figures and Tables

**Table 1:** Average recognition accuracies of different sub-bands (×100%).

Signal	Average recognition accuracy (PE/WPE)	
	SVM	BPNN
a4	0.40/0.64	0.53/0.53
d1	0.58/0.60	0.56/0.67
d2	0.60/0.85	0.65/0.78
d3	0.76/0.84	0.74/0.87
d4	0.48/0.71	0.41/0.54
Undecomposed sEMG	0.86/0.90	0.75/0.82

**Table 2:** Recognition accuracies of d3 and undecomposed sEMG for the seven hand movements (×100%).

Hand movement	d3 (PE/WPE)		Undecomposed sEMG(PE/WPE)	
	SVM	BPNN	SVM	BPNN
Open	0.64/0.71	0.77/1.00	0.87/0.92	1.00/0.62
Close	0.62/0.87	0.69/1.00	1.00/0.83	0.92/0.62
Point	0.71/0.82	0.85/0.92	0.82/1.00	0.85/0.92
Ok	0.78/0.79	0.38/0.85	0.90/0.80	0.54/0.77
Yeah	0.76/0.92	0.92/0.77	0.69/0.92	0.92/1.00
Tripod	0.92/0.89	0.92/0.92	0.92/0.93	0.62/1.00
Grip	0.88/1.00	0.54/0.69	0.80/0.92	0.38/0.85

**Table 3:** Summary of average recognition accuracies (×100%).

Method	Features	SVM	BPNN
Without decomposition	PE	0.86	0.75
	WPE	0.90	0.82

With wavelet decomposition	Wavelet PE feature set (all sub-bands)	1.00	0.97
	WWPE feature set (all sub-bands)	1.00	0.98
	Wavelet PE feature set (single d3 sub-band)	0.57	0.58
	WWPE feature set (single d3 sub-band)	0.75	0.67

**Table 4:** Recognition accuracies of Wavelet PE feature set and WWPE feature set (all sub-bands) for the seven hand movements (×100%).

Hand movement	Wavelet PE feature set		WWPE feature set	
	SVM	BPNN	SVM	BPNN
Open	1.00	1.00	1.00	1.00
Close	1.00	1.00	1.00	1.00
Point	1.00	1.00	1.00	1.00
Ok	1.00	1.00	1.00	1.00
Yeah	1.00	1.00	1.00	1.00
Tripod	1.00	1.00	1.00	1.00
Grip	1.00	1.00	1.00	1.00

**Table 5:** The commonly used time-domain feature set recognition accuracies (×100%).

Hand movement	Open	Close	Point	Ok	Yeah	Tripod	Grip
BP	0.70	0.89	0.85	0.69	0.94	0.82	0.70
SVM	0.84	0.95	0.92	0.85	0.83	0.93	0.77

## Data Availability

The raw/processed data required to reproduce these findings cannot be shared at this time as the data also form part of an ongoing study.

## References

[1] Pal K., Mitra K., Bit A., Bhattacharyya S., Dey A. (2018). Medical signal processing in biomedical and clinical applications. *Journal of Healthcare Engineering*.

[2] Kalani H., Moghimi S., Akbarzadeh A. (2016). Towards an SEMG-based tele-operated robot for masticatory rehabilitation. *Computers in Biology and Medicine*.

[3] Jiang M., Gia T. N., Anzanpour A. IoT-based remote facial expression monitoring system with sEMG signal.

[4] Al Omari F., Hui J., Mei C., Liu G. (2014). Pattern recognition of eight hand motions using feature extraction of forearm EMG signal. *Proceedings of the National Academy of Sciences, India Section A: Physical Sciences*.

[5] Khushaba R. N., Al-Timemy A., Kodagoda S., Nazarpour K. (2016). Combined influence of forearm orientation and muscular contraction on EMG pattern recognition. *Expert Systems with Applications*.

[6] Vujaklija I., Shalchyan V., Kamavuako E. N. (2018). Online mapping of EMG signals into kinematics by autoencoding. *Journal of Neuroengineering and Rehabilitation*.

[7] Samuel O. W., Li X., Geng Y. (2017). Resolving the adverse impact of mobility on myoelectric pattern recognition in upper-limb multifunctional prostheses. *Computers in Biology and Medicine*.

[8] Asogbon M. G., Samuel O. W., Geng Y. (2019). Towards resolving the Co-existing impacts of multiple dynamic factors on the performance of EMG-pattern recognition based prostheses. *Computer Methods and Programs in Biomedicine*.

[9] Geng Y., Ouyang Y., Samuel O. W. (2018). A robust sparse representation based pattern recognition approach for myoelectric control. *IEEE Access*.

[10] Parajuli N., Sreenivasan N., Bifulco P. (2019). Real-time EMG based pattern recognition control for hand prostheses: a review on existing methods, challenges and future implementation. *Sensors*.

[11] Veer K., Sharma T. (2016). A novel feature extraction for robust EMG pattern recognition. *Journal of Medical Engineering & Technology*.

[12] Xing K., Yang P., Huang J., Wang Y., Zhu Q. (2014). A real-time EMG pattern recognition method for virtual myoelectric hand control. *Neurocomputing*.

[13] Samuel O. W., Zhou H., Li X. (2018). Pattern recognition of electromyography signals based on novel time domain features for amputees’ limb motion classification. *Computers & Electrical Engineering*.

[14] Tsai A.-C., Luh J.-J., Lin T.-T. (2015). A novel STFT-ranking feature of multi-channel EMG for motion pattern recognition. *Expert Systems with Applications*.

[15] Al Harrach M., Carriou V., Boudaoud S., Laforet J., Marin F. (2017). Analysis of the sEMG/force relationship using HD-sEMG technique and data fusion: a simulation study. *Computers in Biology and Medicine*.

[16] Qin Z., Jiang Z., Chen J., Hu C., Ma Y. (2019). sEMG-based tremor severity evaluation for Parkinson’s disease using a light-weight CNN. *IEEE Signal Processing Letters*.

[17] Samuel O. W., Asogbon M. G., Geng Y. A novel time-domain descriptor for improved prediction of upper limb movement intent in EMG-PR system. https://www.researchgate.net/deref/http%3A%2F%2Fdx.doi.org%2F10.1109%2FEMBC.2018.8513015.

[18] Alam R.-u, Rhivu S. R., Haque M. Improved gesture recognition using deep neural networks on sEMG.

[19] Yang Q., Wang J. (2016). A wavelet based multiscale weighted permutation entropy method for sensor fault feature extraction and identification. *Journal of Sensors*.

[20] Peng Z. K., Chu F. L. (2004). Application of the wavelet transform in machine condition monitoring and fault diagnostics: a review with bibliography. *Mechanical Systems and Signal Processing*.

[21] Duan F., Dai L., Chang W., Chen Z., Zhu C., Li W. (2015). sEMG-based identification of hand motion commands using wavelet neural network combined with discrete wavelet transform. *IEEE Transactions on Industrial Electronics*.

[22] Subasi A., Alaskandarani A., Abubakir A. A., Qaisar S. M. sEMG signal classification using DWT and bagging for basic hand movements. https://www.researchgate.net/deref/http%3A%2F%2Fdx.doi.org%2F10.1109%2FNCG.2018.8593010.

[23] Gokgoz E., Subasi A. (2015). Comparison of decision tree algorithms for EMG signal classification using DWT. *Biomedical Signal Processing and Control*.

[24] Vavadi H., Ayatollahi A., Mirzaei A. (2010). A wavelet-approximate entropy method for epileptic activity detection from EEG and its sub-bands. *Journal of Biomedical Science and Engineering*.

[25] Eskov V. M., Eskov V. V., Vochmina Y. V., Gorbunov D. V., Ilyashenko L. K. (2017). Shannon entropy in the research on stationary regimes and the evolution of complexity. *Moscow University Physics Bulletin*.

[26] Vuong P. L., Malik A. S., Bornot J. Weighted-permutation entropy as complexity measure for electroencephalographic time series of different physiological states.

[27] Fadlallah B., Chen B., Keil A., Príncipe J. (2013). Weighted-permutation entropy: a complexity measure for time series incorporating amplitude information. *Physical Review E*.

[28] Menon R., Di Caterina G., Lakany H., Petropoulakis L., Conway B. A., Soraghan J. J. (2017). Study on interaction between temporal and spatial information in classification of EMG signals for myoelectric prostheses. *IEEE Transactions on Neural Systems and Rehabilitation Engineering*.

[29] Zahin S., Iqbal O., Fattah S. A., Shahnaz C. Hand action classification via wavelet reconstruction and sub-frame based feature extraction.

[30] Chen Z., Liu Y., Liu Z., Tang H. The selection of wavelet function in singular signal detection.

[31] Xia K., He S., Tan Y., Jiang Q., Xu J., Yu W. (2019). Wavelet packet and support vector machine analysis of series DC ARC fault detection in photovoltaic system. *IEEJ Transactions on Electrical and Electronic Engineering*.

[32] Bandt C., Pompe B. (2002). Permutation entropy: a natural complexity measure for time series. *Physical Review Letters*.

